# Clinical Characteristics of Overweight Patients With Acute Exacerbation Chronic Obstructive Pulmonary Disease (AECOPD)

**DOI:** 10.1111/crj.70001

**Published:** 2024-08-26

**Authors:** Yuxin Gong, Fawang Du, Yu Yao, Hanchao Wang, Xiaochuan Wang, Wei Xiong, Qin Wang, Gaoyan He, Linlin Chen, Heng Du, Juan Yang, Brent A. Bauer, Zhongruo Wang, Huojin Deng, Tao Zhu

**Affiliations:** ^1^ Department of Pulmonary and Critical Care Medicine Zhujiang Hospital, Southern Medical University Guangzhou Guangdong China; ^2^ Department of Pulmonary and Critical Care Medicine Suining Central Hospital Suining Sichuan China; ^3^ Department of Pulmonary and Critical Care Medicine Second Affiliated Hospital of Chongqing Medical University Chongqing China; ^4^ Department of Gastroenterology Medicine Suining Central Hospital Suining Sichuan China; ^5^ Division of General Internal Medicine Mayo Clinic Rochester Minnesota USA; ^6^ Department of Mathematics University of California Davis USA

**Keywords:** chronic obstructive pulmonary disease (COPD), hypertension, nomogram, overweight, type 2 diabetes (T2DM)

## Abstract

**Introduction:**

Low body weight in patients with COPD is associated with a poor prognosis and more comorbidities. However, the impact of increased body weight in patients with COPD remains controversial. The aim of this study was to explore the clinical features of overweight patients with AECOPD.

**Methods:**

In this multicenter cross‐sectional study, a total of 647 AECOPD patients were recruited. Finally, 269 normal weight and 162 overweight patients were included. Baseline characteristics and clinical and laboratory data were collected. The least absolute shrinkage and selection operator (LASSO) regression was performed to determine potential features, which were substituted into binary logistic regression to reveal overweight‐associated clinical features. The nomogram and its associated curves were established to visualize and verify the logistic regression model.

**Results:**

Six potential overweight‐associated variables were selected by LASSO regression. Subsequently, a binary logistic regression model identified that the rates of type 2 diabetes (T2DM) and hypertension and levels of lymphocytes (LYM)%, and alanine aminotransferase (ALT) were independent variables of overweight in AECOPD patients. The C‐index and AUC of the ROC curve of the nomogram were 0.671 and 0.666, respectively. The DCA curve revealed that the nomogram had more clinical benefits if the threshold was at a range of 0.22~0.78.

**Conclusions:**

Collectively, we revealed that T2DM and hypertension were more common, and LYM% and ALT were higher in AECOPD patients with overweight than those with normal weight. The result suggests that AECOPD patients with overweight are at risk for additional comorbidities, potentially leading to worse outcomes.

## Graphical abstract

Six potential overweight‐associated variables were selected by LASSO regression. This study found that T2DM and hypertension were more common, and LYM% and ALT were higher in AECOPD patients with overweight than those with normal weight.

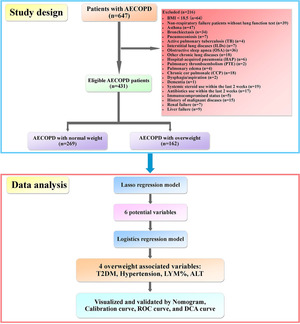

## 1 Introduction

Chronic obstructive pulmonary disease (COPD) is the most common respiratory disease and the third leading cause of death globally [1]. In 2010, it was estimated that the worldwide general prevalence of COPD was 11.7% (95% CI: 8.4%~15.0%), indicating that around 384 million people suffered from COPD [2]. Concurrently, overweight and obesity are major health burdens in the world, which are still increasing and getting worse in recent decades [3]. Mounting evidence suggests that overweight plays an adverse role in almost all physiological functions, as well as a critical risk factor for many diseases, such as cardiovascular disease, diabetes, cancers, and asthma [4, 5, 6, 7].

However, the impact of overweight in lung functions and COPD remains unclear. Most studies showed that overweight was associated with reduced lung function in the general population [8]. In a meta‐analysis, Forno et al. found that overweight/obesity was negatively correlated with most lung function parameters, including FEV1%, FVC%, FEV1/FVC%, FEF_25–75_%, TLC%, RV%, and FRC%, across all age groups, regardless of asthma status [8]. Lambert et al. showed that obesity was independently associated with worse respiratory‐specific and general quality of life (QOL), decreased 6‐min walk distance (6MWD), increased Modified Medical Research Council (MMRC) scores, and greater odds of severe acute exacerbation in COPD patients [9]. On the other hand, Smulders, van der Aalst, and Neuhaus demonstrated that the exacerbation frequency requiring hospitalization and the time to readmission were markedly lower in COPD patients with obesity compared to those with normal weight [10]. Wu et al. reported that lung functions were positive, and blood eosinophils count and inflammatory parameters were negatively correlated with BMI in COPD patients [11]. Meanwhile, they identified that increased BMI was also associated with less exacerbation and hospitalization. Additionally, in a retrospective study from Korea, Lee et al. revealed that the presence of overweight was positively associated with FEV1/FVC% in general adult [12].

Although a variety of diversities were observed between overweight and normal weight in COPD patients, the differences in clinical features, especially laboratory parameters, were seldom explored in patients with AECOPD. Meanwhile, identifying modifiable risk factor of overweight would potentially reduce adverse events in AECOPD patients at an early stage. Simultaneously, we noticed many overweight‐related differences in daily clinical practices. Therefore, the purpose of this study was to investigate the clinical features of overweight hospitalized AECOPD patients. Additionally, a nomogram and its associated curves were used to visualize and verify the logistic regression model, which will potentially assist physicians in making better clinical decisions.

## 2 Materials and Methods

### 2.1 Study Design and Population

According to our previous studies [13, 14], this multicenter cross‐sectional study was performed, and ethnic was approved by our hospitals. Meanwhile, the standard management was provided in this study.

### 2.2 Sample Size Determinations

According to our previous studies [14, 15], the sample size was calculated. Based on the previous study [16], the ratio of normal weight to overweight and obese in Chinese COPD patients is around 2:1. Therefore, a minimum of 396 participants (264 normal weight participants and 132 overweight participants) was required with effect size = 0.3, power = 0.8, α = 0.05, and allocation ratio = 2:1. Meanwhile, additional 20% patients were enrolled.

### 2.3 Inclusion and Exclusion Criteria

**FIGURE 1:**
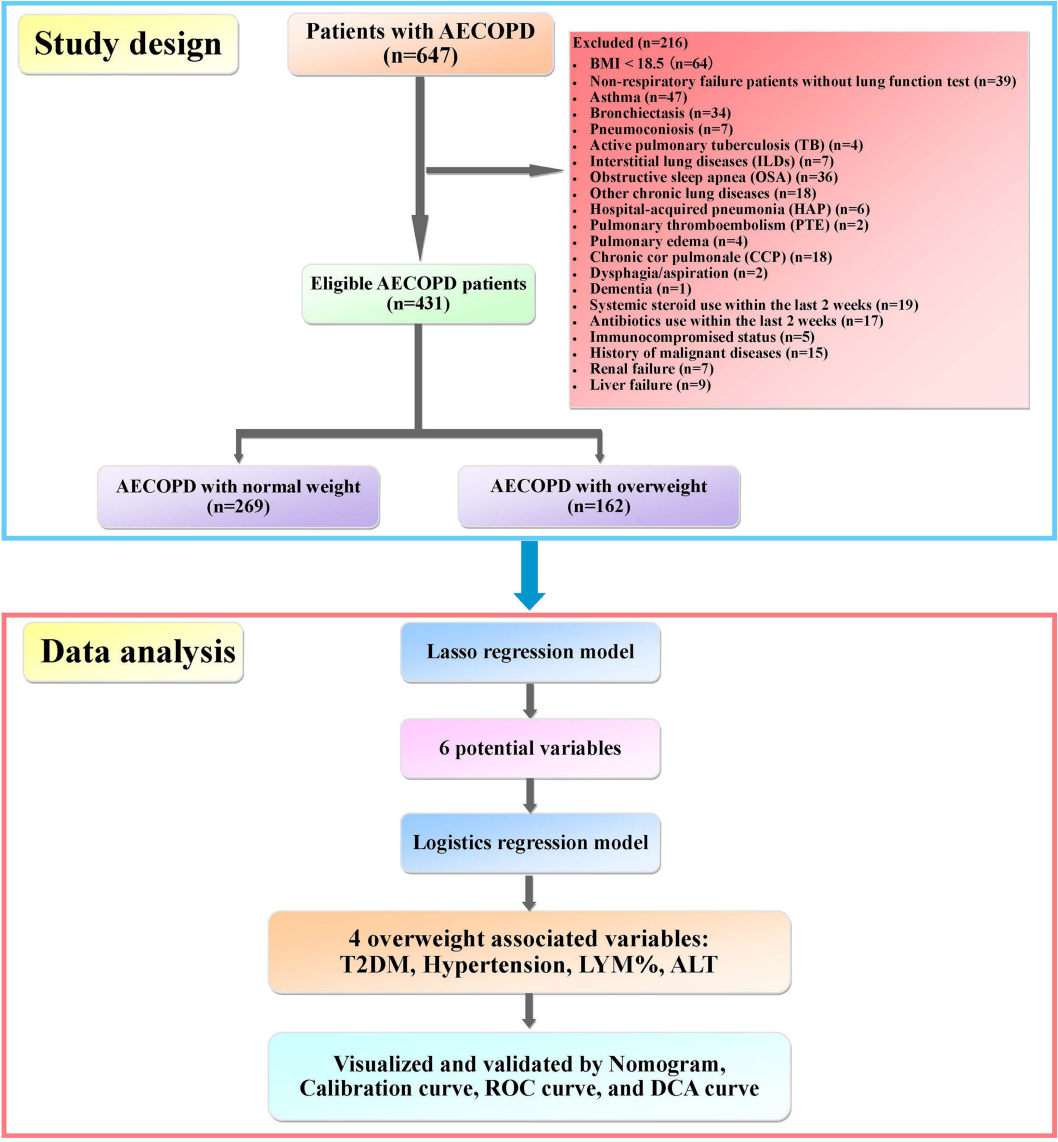
Summary of study design and data analysis.

The inclusion criterion was hospitalized AECOPD. Exclusion criteria are shown in Figure 1. Briefly, a total of 647 patients with hospitalized AECOPD were enrolled. Among them, 216 were excluded. The final accrual included 269 who were normal weight (BMI 18.5~23.9), and 162 who were overweight (BMI 24~30) (Figure 1). Additionally, overweight, normal weight, and low weight were defined as 30 > BMI ≥ 24 kg/m^2^, 18.5 ~ 23.9 kg/m^2^, and < 18.5 kg/m^2^, respectively [16].

### 2.4 Data Collection

According to our previous studies [13, 14, 15, 17], demographics, clinical data, laboratory data, and chest HRCT data of AECOPD patients were collected.

### 2.5 Statistical Analysis

According to our previous studies [14, 15, 17], statistical analysis was performed. Briefly, data were analyzed using R software version 4.1.2. Variance inflation factor (VIF) was utilized to determine variable multicollinearities. VIF ≥ 10 implied high multicollinearity, which was excluded in subsequent analysis. The potential variables associated with overweight were first selected using LASSO regression. Then, the binary logistic regression model was built using these LASSO regression‐selected variables. Furthermore, the binary logistic regression model was verified and visualized by nomogram and its associated curves. *p* < 0.05 indicated a significant difference.

## 3 Results

### 3.1 Demographic Data

In this study, 647 AECOPD patients were recruited. Finally, 269 (62.4%) patients were normal weight, and 162 (37.6%) patients were overweight (Figure 1). Older age, less smoking, and higher rates of hypertension and T2DM were found in overweight patients compared to normal weight patients (Table 1).

**TABLE 1:** Demographic data of patients with AECOPD (*n* = 431).

	Normal weight (*n* = 269)	Overweight (*n* = 162)	Statistical values	*p*
Sex (male, *n* (%))	216 (80.3%)	118 (72.8%)	3.22	0.073
Age (years)^a^	69.78 ± 9.03	71.40 ± 8.31	−1.98	0.048
Body mass index (BMI)^a^	21.26 ± 1.60	26.21 ± 1.69	−17.40	< 0.001
Smoking			−2.71	0.007
Nonsmoking	87 (32.3%)	72 (44.4%)		
Ex‐smoking	61 (22.7%)	36 (22.2%)		
Current smoking	121 (45.0%)	54 (33.3%)		
GOLD stages			−1.50	0.133
Stage I: mild (≥ 80%)	31 (11.5%)	24 (14.8%)		
Stage II: moderate (50%–79%)	90 (33.5%)	62 (38.3%)		
Stage III: severe (30%–49%)	76 (28.3%)	40 (24.7%)		
Stage IV: very severe (< 30%) without respiratory failure	30 (11.2%)	11 (6.8%)		
Respiratory failure	42 (15.6%)	25 (15.4%)		
Underlying diseases/comorbidities				
Pleural effusion	5 (1.9%)	2 (1.2%)	0.25	0.620
Community‐acquired pneumonia (CAP)	112 (41.6%)	72 (44.4%)	0.33	0.568
Coronary artery disease (CAD)	39 (14.5%)	34 (21.0%)	3.03	0.082
Hypertension	93 (34.6%)	79 (48.8%)	8.49	0.004
Type 2 diabetes (T2DM)	29 (10.8%)	38 (23.5%)	12.38	< 0.001
Atrial fibrillation (Af)	5 (1.9%)	5 (3.1%)	0.67	0.412
Mechanical ventilation (MV)			−1.07	0.286
Nonventilation	257 (95.5%)	158 (97.5%)		
Noninvasive positive pressure ventilation (NIPPV)	10 (3.7%)	4 (2.5%)		
Invasive positive pressure ventilation (IPPV)	2 (0.7%)	0 (0.0%)		

^a^ Continuous data without normal distribution.

### 3.2 Clinical Manifestations and Laboratory Results

Compared to AECOPD patients with normal weight, the level of alanine aminotransferase (ALT) was noticeably higher in AECOPD patients with overweight (Table 2).

**TABLE 2:** Clinical features and laboratory data of patients with AECOPD (*n* = 431).

	Normal weight (*n* = 269)	Overweight (*n* = 162)	Statistical value	*p*
Fever	23 (8.6%)	12 (7.4%)	0.18	0.674
White blood cells (WBC) (×10^9^/L)	7.65 ± 3.28	7.71 ± 3.29	−0.19	0.847
Neutrophils (NS) (×10^9^/L)	6.00 ± 3.18	5.91 ± 3.24	0.28	0.779
Lymphocytes (LYM) (×10^9^/L)	1.44 ± 0.73	1.56 ± 0.72	−1.71	0.088
Eosinophils (EOS) (×10^9^/L)	0.21 ± 0.23	0.24 ± 0.27	−1.22	0.224
NS%	76.13 ± 10.74	74.17 ± 11.42	1.79	0.074
LYM%	20.85 ± 9.68	22.36 ± 9.97	−1.55	0.122
EOS%	3.02 ± 3.07	3.47 ± 3.63	−1.39	0.166
Neutrophil‐to‐lymphocyte ratio (NLR)	5.08 ± 3.82	4.71 ± 4.00	0.96	0.339
Platelets (PLTs) (×10^9^/L)	204.02 ± 66.65	200.54 ± 64.85	0.53	0.596
Red blood cells (RBCs) (×10^9^/L)	4.48 ± 0.57	4.45 ± 0.55	0.56	0.579
Hemoglobin (Hb) (g/L)	134.88 ± 15.21	132.71 ± 15.70	1.42	0.157
Procalcitonin (PCT) (ng/mL)	0.21 ± 0.40	0.22 ± 0.61	−0.29	0.775
C‐reaction protein (CRP) (mg/mL)	24.27 ± 35.34	24.44 ± 42.02	−0.05	0.964
Erythrocyte sedimentation rate (ESR) (mm/first hour)	21.53 ± 17.16	24.54 ± 21.57	−1.60	0.110
Air blood gas (ABG)				
PH	7.43 ± 0.04	7.43 ± 0.04	−0.92	0.360
PaCO2 (mmHg)	41.39 ± 7.68	41.10 ± 8.03	0.37	0.710
PaO2 (mmHg)	76.20 ± 14.26	75.97 ± 12.54	0.17	0.865
Actual base (AB) (mmol/L)	26.80 ± 3.38	26.86 ± 3.23	−0.20	0.843
Standard base (SB) (mmol/L)	26.57 ± 2.20	26.69 ± 2.36	−0.51	0.609
Anion gap (AG)	10.97 ± 4.19	10.92 ± 4.15	0.12	0.906
Albumin (ALB) (g/L)	38.82 ± 4.29	39.00 ± 3.81	−0.44	0.658
Blood urea nitrogen (BUN) (mmol/L)	6.07 ± 2.20	6.29 ± 2.04	−1.03	0.306
Creatinine (Cr) (μmol/L)	78.94 ± 23.48	78.19 ± 25.37	0.31	0.757
Alanine aminotransferase (ALT) (U/L)	20.35 ± 16.88	27.36 ± 27.23	−3.30	0.001
Aspartate aminotransferase (AST) (U/L)	23.60 ± 18.14	26.64 ± 22.39	−1.54	0.124
Total bilirubin (TBIL) (μmol/L)	10.24 ± 4.95	10.48 ± 4.83	−0.48	0.629
Indirect bilirubin (IBIL) (μmol/L)	5.96 ± 3.09	6.12 ± 2.89	−0.52	0.602
Direct bilirubin (DBIL) (μmol/L)	4.28 ± 2.44	4.36 ± 2.50	−0.32	0.747
Random blood glucose (RBG) (mmol/L)	6.96 ± 2.84	6.98 ± 2.67	−0.06	0.953

### 3.3 LASSO Regression Analysis

**FIGURE 2:**
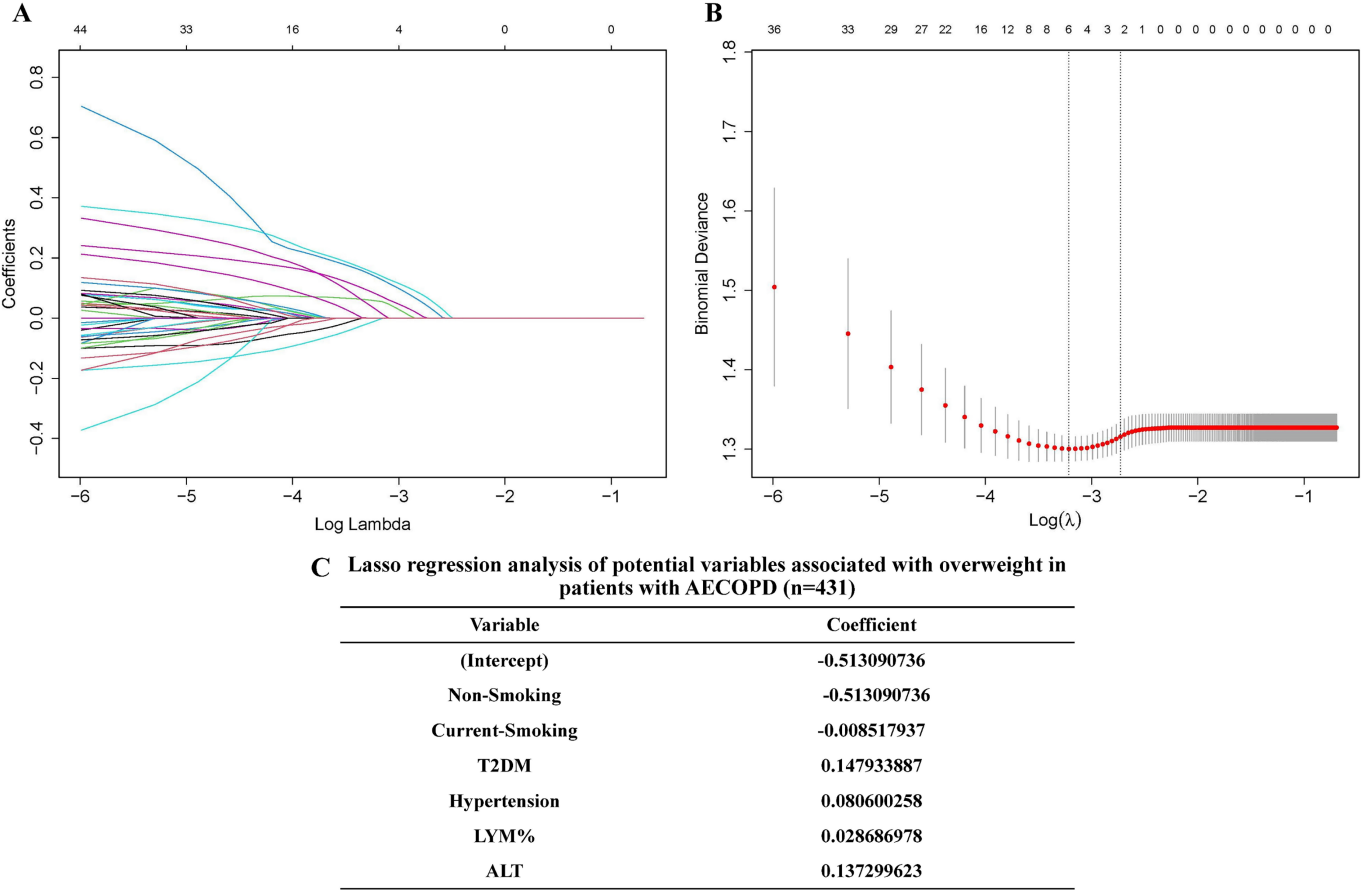
Potential variables associated with overweight in AECOPD patients were selected by the LASSO regression model. (A) LASSO coefficient profiles for all variables. (B) Identification of the optimal penalization coefficient (λ) in the LASSO model, which was carried out by 10‐fold cross‐validation by minimum criteria and 1‐SE (standard error) criteria. Left line: the minimum error; right line: the cross‐validated error within one standard error of the minimum. (C) LASSO coefficient values of six potential variables.

LASSO regression was utilized to decrease data dimension and select overweight‐associated potential variables in AECOPD patients (Figure 2A,B). We found that six variables were with nonzero coefficients, including nonsmoking, current smoking, T2DM, hypertension, LYM%, and ALT (Figure 2C).

### 3.4 Binary Logistic Regression Analysis

The binary logistic regression model was established by six LASSO regression‐selected potential variables. Then, we found that T2DM, hypertension, LYM%, and ALT were independently associated with overweight in AECOPD patients (Table 3). Meanwhile, the C‐index of this logistic regression was 0.671 (95% CI: 0.618~0.723).

**TABLE 3:** Binary logistic regression analysis of independent variables associated with overweight in patients with AECOPD (*n* = 431).

	OR	OR 95% CI	Sig.
(Intercept)	0.16	0.08~0.32	< 0.0001
Nonsmoking	1.24	0.72~2.15	0.4414
Current smoking	0.73	0.42~1.27	0.2726
T2DM	2.37	1.35~4.16	0.0027
Hypertension	1.68	1.09~2.58	0.0181
LYM%	1.03	1.01~1.05	0.0072
ALT	1.02	1.01~1.03	0.0027

Abbreviations: ALT: alanine aminotransferase; LYM: lymphocytes; T2DM: type 2 diabetes.

### 3.5 Nomogram and Its Associated Curves Were Performed to Visualize and Verify the Binary Logistic Regression Model

**FIGURE 3:**
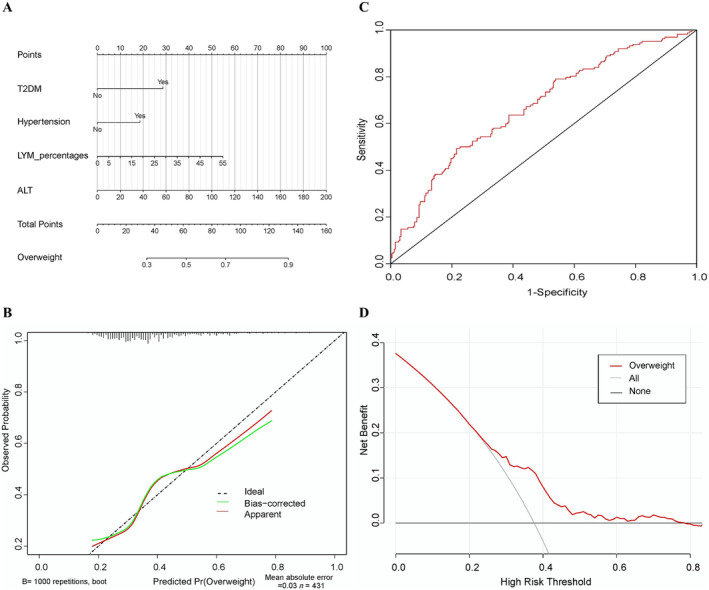
The nomogram for predicting overweight in AECOPD patients. A nomogram was used to visualize and validate the binary logistic regression model. (A) Nomogram. The total point of a specific patient is the sum of individual variable points. The predicted probability of overweight is on the overweight scale, which corresponds to the total point scale. (B) Calibration curves. The ideal line is the nomogram reference line; the apparent line is the actual probability of each patient in our study; the bias‐corrected line is adjusted by bootstrap with 1000 resamples. The length of the vertical lines at the top of the plot represents the number of patients. (C) ROC curve. (D) DCA curve. None line: the assumption that all patients had no overweight. All line: the assumption that all patients had overweight. Red line: the nomogram.

A nomogram was established, based on the aforementioned logistic regression model (Figure 3A). The calibration curve with 1000 bootstrap demonstrated that both the apparent line and the bias‐corrected line were close to the ideal line with mean absolute error (MAE) = 0.03 (Figure 3B). The AUC of the ROC curve was 0.666 (95% CI: 0.613~0.719) (Figure 3C). The DCA curve showed that when the threshold was between 0.22 and 0.78, this nomogram, to predict the overweight probability, took more net benefit than the scheme, indicating well clinical applicability of our model (Figure 3D).

## 4 Discussion

In this multicenter cross‐sectional study, 647 patients with AECOPD were enrolled. Ultimately, 269 subjects with normal weight (BMI 18.5~23.9) and 162 subjects with overweight (BMI 24~30) were included. Meanwhile, demographics, spirometry test, laboratory data (blood routine, ABG, renal and liver functions, and inflammatory parameters), and chest HRCT data were collected. Then, six variables, including nonsmoking, current smoking, T2DM, hypertension, LYM%, and ALT, were selected by LASSO regression, which were potentially relevant to overweight in AECOPD patients. Subsequently, the binary logistic regression model was built using six aforementioned variables, revealing that T2DM, hypertension, LYM%, and ALT were independently associated with overweight in patients with AECOPD. Furthermore, nomogram and its associated curves, including calibration curve, ROC curve, and DCA curve, were performed to visualize and verify the binary logistic regression model. We found that the C‐index and AUC of the ROC curve were 0.671 (95% CI: 0.618~0.723) and 0.666 (95% CI: 0.613~0.719), respectively. The MAE of the calibration curve was 0.03. Additionally, the DCA curve demonstrated that the nomogram with a threshold from 0.22 to 0.78 yielded more net benefit than the scheme, implying a good performance to predict overweight in AECOPD patients. Thus, the results of nomogram and its associated curves validated the accuracy, reliability, and clinical use of this binary logistic regression model. Therefore, these results imply that T2DM, hypertension, excessive systemic inflammation, and abnormal liver function are the independent underlying etiologies and clinical features for AECOPD patients with overweight.

Obesity and overweight have become the major public health concerns. It is estimated that approximately 30%~40% of the global adult population is suffering from them [18]. Obesity is a chronic disease that affects nearly all body functions, increasing the risk of T2DM, depression, dyslipidemia, many types of cancer, and cardiovascular diseases [4, 7, 18]. Meanwhile, COPD is a highly heterogenous respiratory disease. Precise medicine and individual therapy have been the trend in COPD management. Several studies revealed that overweight COPD patients were a unique subgroup, having relatively specific clinical features [19, 20, 21]. However, the role of overweight in COPD is still controversial [10]. In an observative study, 342 COPD patients were recruited [21]. Then, three clusters of COPD patients were identified, which presented distinct clinical features and outcomes. Among them, one subgroup (greater prevalence of overweight) had higher rates of cardiovascular disease and T2DM, and more severe systemic inflammation. In the follow‐up, this subgroup had more cardiovascular disease‐associated admissions, indicating worse prognosis and outcomes. In Genetic Epidemiology of COPD (COPDGene), a multicenter prospective cohort study, 3631 COPD participants were enrolled [9]. Study results showed that overweight was independently associated with poor outcomes, such as worse QOL, reduced 6MWD, and increased mMRC scores. Simultaneously, several studies reported that overweight might provide some benefits in COPD patients [4, 11, 18, 22]. In a multicenter retrospective cohort in Taiwan, 1096 COPD patients from 12 hospitals were included [22]. In this study, overweight was independently associated with a lower frequency of COPD exacerbations. In a retrospective real‐world study, 774 patients with COPD in China were screened [11]. It was found that increased BMI was positively correlated with spirometry results, including FEV1, PEF, and FEF25/50/75, diffusing capacity of carbon monoxide (DLCO), while negatively correlated with CRP, blood EOS, the systemic corticosteroid dosage, the length of hospital stay, and the frequency of exacerbation and hospitalization. DeLapp et al. demonstrated that overweight was a key predictor of reduced mortality at 1 year and 6 months in patients with AECOPD [23]. In a retrospective cohort study, Lainscak et al. also reported that overweight was an independent predictor of better long‐term survival in AECOPD patients [24]. However, these previous studies were more focused on the clinical symptoms and subjective parameters, such as mMRC scores, 6MWD, and QOL, as well as long‐term prognosis, rather than objective characteristics and parameters, especially laboratory results. Otherwise, the results of the retrospective study, in which data was from a medical history record, are less accurate than a well‐designed prospective study in general. Thus, in the current study, demographics, underlying diseases, comorbidities, and clinical manifestations also were recorded. More importantly, comprehensive laboratory data, lung functions, and chest HRCT images were all collected and analyzed to explore the clinical features, particularly objective clinical parameters, risk factors, and etiology of overweight in AECOPD patients.

Based on univariate analysis, five variables with significant differences (*p* < 0.05) between the two groups were observed (Tables 1 and 2). LASSO regression, a regression‐based methodology, can decline covariance among multiple factors, decrease the possibility of overfitting, remove unnecessary covariates, and minimize the multicollinearity in variables [15]. Then, LASSO regression has been widely used as an accurate and effective method for variable selection and regularization in clinical studies [15, 25]. In the current study, six potential overweight‐associated variables were identified by LASSO regression (Figure 2). Subsequently, among these six variables, binary logistic regression revealed that T2DM, hypertension, LYM%, and ALT were independently associated with overweight in patients with AECOPD (Table 3).

It is well‐known that overweight/obesity is the most important risk factor for diabetes and hypertension in the general population [21, 26, 27, 28]. Meanwhile, several studies also demonstrated that both diabetes and hypertension were common comorbidities in COPD patients, which were linked to prognosis and outcomes [29, 30]. In a retrospective cross‐sectional study in the Netherlands, 1654 COPD patients were included [31]. It was found that the prevalence of diabetes, hypertension, atrial fibrillation, and congestive heart failure in COPD patients with overweight was markedly higher than in COPD patients without overweight. Then, in another cross‐sectional study in Belgium and the Netherlands, 527 stable COPD patients were classified into three phenotypes using Ward's cluster analysis combined with multiple correspondence analyses (MCAs) and principle component analysis (PCA) [19]. Among them, one phenotype had more male, older age, higher BMI, and higher rates of diabetes and cardiovascular comorbidities. Furthermore, in a prospective observation study, 213 COPD patients were clustered into five subgroups [20]. Of these, patients in the metabolic subgroup also had more males, higher rates of obesity, diabetes, hypertension, dyslipidemia, and atherosclerosis. Consistent with these studies, our data identified that T2DM and hypertension were independently associated with overweight in AECOPD patients. These results indicate that overweight/obesity increases the risks of T2DM and hypertension not just in the general population, but also in COPD patients, which may account for worse outcomes and prognosis in COPD. Since age plays a significant role in the pathogenesis and development of COPD, hypertension, and diabetes, the older age was observed in AECOPD patients with overweight; however, age was not selected by LASSO regression. Additionally, chronic systemic inflammation is essential for the pathogenesis of COPD and diabetes [1, 29, 32]. It was found that some key inflammatory mediators of COPD, particularly IL‐6, TNF‐α, and TGF‐β, also play hub roles in insulin resistance and diabetes [30, 32, 33]. Furthermore, the recent studies showed that glucagon‐like peptide‐1 receptor agonist (GLP‐1RA), a widely used antidiabetes drug, was also a potential and promising therapy for chronic airway diseases, including COPD and asthma [32, 34, 35]. GLP‐1RA also has the benefits of weight loss, blood pressure reduction, and cardiovascular protection [36]. Therefore, both preclinical and clinical studies are warranted to explore their internal connections and underlying mechanisms in the future.

Simultaneously, as aforementioned, COPD is characterized by persistent airway inflammation and low‐degree systemic inflammation, which causes a variety of extrapulmonary effects and comorbidities [20], such as diabetes, hypertension, atherosclerosis, skeletal muscle dysfunction, osteoporosis, and pulmonary fibrosis [14, 15, 29, 37]. It was found that COPD combined with diabetes had worse prognosis and clinical outcomes [26, 27, 38]. Furthermore, cellular senescence, systemic inflammation, oxidative stress, hypoxemia, and hyperglycemia play roles both in the pathogenesis of diabetes and COPD [26, 38, 39, 40]. Tumor necrosis factor‐a receptors 1 (TNF‐R1) and 2 (TNF‐R2) in the blood of the metabolic subgroup were markedly higher than other subgroups. Meanwhile, Peres et al. showed that reduced expressions of CD25+, HLA‐DR, and CCr5 on the cell surface of T‐cells and decreased IL‐2 and increased IL‐6 and INF‐γ in blood were observed in COPD subjects with overweight compared to those with normal weight [41]. Consistent with these findings, our data also demonstrated that the trend of increasing LYM% in blood was independently associated with overweight in AECOPD patients, implying that overweight/obesity further worsens systemic inflammation in COPD patients. However, the roles of lymphocytes and their different subsets in COPD‐obesity interactions are important and interesting issues for future research. Then, the role of obesity in lymphocyte‐associated airway inflammation in COPD is a critical and interesting area to explore.

A number of studies revealed that overweight is a key risk factor of nonalcoholic fatty liver disease (NAFLD), which can induce liver chronic inflammation and injury, subsequently, leading to mild to moderately asymptomatic elevation of abnormal liver enzyme levels, particularly ALT and aspartate aminotransferase (AST) [42, 43, 44]. Meanwhile, it is reported that ALT elevations were more common than elevations of AST in NAFLD patients [42, 45]. Consistently, our data also found that the trend of increasing ALT was independently associated with overweight in AECOPD patients. However, the interactions and mutual impacts of COPD, obesity, smoking, NAFLD, and metabolic syndrome are very complicated [42, 46]. Therefore, more variables and data, such as ultrasound, lipid metabolism, and viral hepatitis laboratory parameters, should be included in future study to explore their connections.

Additionally, the nomogram and its associated curves were built to visualize and verify the binary logistic regression model. The C‐index of this logistic regression was 0.671. The AUC of the ROC curve was 0.666. The ideal line and apparent line were very close in the calibration curve with an MAE of 0.03. These data suggest the relatively high sensitivity and specificity of the nomogram and binary logistic regression model. Simultaneously, the DCA curve was performed to evaluate the clinical utility of nomograms, which demonstrated that our nomogram had a good performance to predict overweight with the threshold range of 22%~78%. Collectively, these findings indicate the high accuracy and uniformity of this nomogram, confirming the reliability of our binary logistic regression results.

In the current study, comprehensive clinical data, including underlying diseases, comorbidities, lung function, and laboratory data, were obtained, which was one of the major strengths of this study. Chest HRCT scan was performed in each patient, which was critical to diagnose and exclude other lung diseases, such as bronchiectasis, ILDs, and active pulmonary TB, and to identify the underlying diseases and comorbidities of AECOPD, promoting the accuracy of the results. Meanwhile, LASSO regression was used to reduce the multicollinearity in variables and select potential overweight‐associated variables, which is more efficient and accurate than univariate analysis. Additionally, the nomogram and its associated curves were obtained to visualize and validate the logistic regression model. However, due to the cross‐sectional design, the association between overweight and prognosis was not investigated in AECOPD patients.

## 5 Conclusions

Collectively, our data identified that T2DM, hypertension, and the trend of increasing LYM% and ALT were independently associated with overweight in AECOPD patients. The results suggest that overweight/obesity also is a critical risk factor and etiology of hypertension and diabetes in COPD patients. COPD‐induced systemic inflammation is further enhanced by overweight. However, the role of different subsets of lymphocytes in obesity‐COPD inflammation interaction remains unclear. Meanwhile, diverse etiology‐induced acute exacerbation had specific immunological response and clinical presentations in COPD patients. Some studies reported that air pollution, virus, and bacteria‐induced inflammation and clinical outcomes were different in AECOPD patients [47, 48, 49]. Therefore, individual etiology‐associated immunological reaction and clinical features in patients with AECOPD are a promising area and valuable topic which needs further investigation. Additionally, the underlying mechanism of the trend of increasing ALT and liver dysfunction in overweight and AECOPD is still unknown. Then, further prospective study is warranted to investigate this issue. This study provides evidence that overweight is a unique COPD subgroup with significantly more comorbidities and severe inflammation, which potentially leads to poor prognosis and outcomes. This also highlights the importance of weight control in patients with COPD. However, these findings should be validated by future studies with larger sample sizes. Otherwise, more variables, such as lipid and glucose metabolism parameters and subtypes of lymphocytes, also are worth to investigate in future studies to explore their underlying mechanism and their relationships.

## Author Contributions

Tao Zhu and Huojin Deng conceived the study design. Yuxin Gong, Fawang Du, Yu Yao, Xiaochuan Wang, Wei Xiong, Qin Wang, Gaoyan He, Linlin Chen, and Heng Du collected, collated, and checked data. Yuxin Gong, Fawang Du, Yu Yao, and Juan Yang drafted and revised the manuscript with the help of Brent A. Bauer and Tao Zhu. Hanchao Wang, Tao Zhu, Juan Yang, and Zhongruo Wang analyzed and interpreted the data. Yuxin Gong, Fawang Du, Yu Yao, and Hanchao Wang contributed equally to this work. All authors read and approved the final manuscript.

## Ethics Statement

This study adheres to the amended Declaration of Helsinki criteria. The research protocol was approved by the Ethics Committee of ZhuJiang Hospital of Southern Medical University (No. 2022‐KY‐137), Suining Central Hospital (NO.LLSLH20220046), and Second Affiliated Hospital of Chongqing Medical University (No. 2019–23). Informed consent was obtained from each subject.

## Conflicts of Interest

The authors declare no conflicts of interest.

## Supporting information


**Data S1** Supplementary information.

## Data Availability

The data that support the findings of this study are available from the corresponding author upon reasonable request.
