# Supplementary material for: Clinical Characteristics of Overweight Patients With Acute Exacerbation Chronic Obstructive Pulmonary Disease (AECOPD)
Source: Clin Respir J. 2024 Aug 26;18(8):e70001. doi: 10.1111/crj.70001 (PMC11347388; doi:10.1111/crj.70001)
Supplement: Supplementary file 1 — Data S1 Supplementary information. [file CRJ-18-e70001-s001.pdf]

TRIPOD Checklist: Prediction Model Development and Validation

Section/Topic	Item		Checklist Item	Section
**Title and abstract**				
Title	1	D;V	Identify the study as developing and/or validating a multivariable prediction model, the target population, and the outcome to be predicted.	Title
Abstract	2	D;V	Provide a summary of objectives, study design, setting, participants, sample size, predictors, outcome, statistical analysis, results, and conclusions.	Abstract para 1-4
**Introduction**				
Background and objectives	3a	D;V	Explain the medical context (including whether diagnostic or prognostic) and rationale for developing or validating the multivariable prediction model, including references to existing models.	Intro para 1-2
	3b	D;V	Specify the objectives, including whether the study describes the development or validation of the model or both.	Intro para 3
**Methods**				
Source of data	4a	D;V	Describe the study design or source of data (e.g., randomized trial, cohort, or registry data), separately for the development and validation data sets, if applicable.	Methods para 1, Figure 1
	4b	D;V	Specify the key study dates, including start of accrual; end of accrual; and, if applicable, end of follow-up.	Methods para 1, Figure 1
Participants	5a	D;V	Specify key elements of the study setting (e.g., primary care, secondary care, general population) including number and location of centres.	Methods para 1
	5b	D;V	Describe eligibility criteria for participants.	Methods para 3
	5c	D;V	Give details of treatments received, if relevant.	NA
Outcome	6a	D;V	Clearly define the outcome that is predicted by the prediction model, including how and when assessed.	Methods para 3
	6b	D;V	Report any actions to blind assessment of the outcome to be predicted.	NA
Predictors	7a	D;V	Clearly define all predictors used in developing or validating the multivariable prediction model, including how and when they were measured.	Methods para 4
	7b	D;V	Report any actions to blind assessment of predictors for the outcome and other predictors.	NA
Sample size	8	D;V	Explain how the study size was arrived at.	Methods para 2
Missing data	9	D;V	Describe how missing data were handled (e.g., complete-case analysis, single imputation, multiple imputation) with details of any imputation method.	NA
Statistical analysis methods	10a	D	Describe how predictors were handled in the analyses.	Methods para 5
	10b	D	Specify type of model, all model-building procedures (including any predictor selection), and method for internal validation.	Methods para 5
	10c	V	For validation, describe how the predictions were calculated.	Methods para 5
	10d	D;V	Specify all measures used to assess model performance and, if relevant, to compare multiple models.	Methods para 5
	10e	V	Describe any model updating (e.g., recalibration) arising from the validation, if done.	Methods para 5
Risk groups	11	D;V	Provide details on how risk groups were created, if done.	NA
Development vs. validation	12	V	For validation, identify any differences from the development data in setting, eligibility criteria, outcome, and predictors.	NA
**Results**				
Participants	13a	D;V	Describe the flow of participants through the study, including the number of participants with and without the outcome and, if applicable, a summary of the follow-up time. A diagram may be helpful.	Figure 1
	13b	D;V	Describe the characteristics of the participants (basic demographics, clinical features, available predictors), including the number of participants with missing data for predictors and outcome.	Table 1-2
	13c	V	For validation, show a comparison with the development data of the distribution of important variables (demographics, predictors and outcome).	Table 1
Model development	14a	D	Specify the number of participants and outcome events in each analysis.	Results para 1
	14b	D	If done, report the unadjusted association between each candidate predictor and outcome.	NA
Model specification	15a	D	Present the full prediction model to allow predictions for individuals (i.e., all regression coefficients, and model intercept or baseline survival at a given time point).	Results para 4, Table 3
	15b	D	Explain how to the use the prediction model.	Results para 5, Tables 3, Figure 2
Model performance	16	D;V	Report performance measures (with CIs) for the prediction model.	Results para 5 , Tables 3
Model-updating	17	V	If done, report the results from any model updating (i.e., model specification, model	NA

			performance).	
**Discussion**				
Limitations	18	D;V	Discuss any limitations of the study (such as nonrepresentative sample, few events per predictor, missing data).	Discussion para 9
Interpretation	19a	V	For validation, discuss the results with reference to performance in the development data, and any other validation data.	Discussion para 2-5
	19b	D;V	Give an overall interpretation of the results, considering objectives, limitations, results from similar studies, and other relevant evidence.	Discussion para 1-9
Implications	20	D;V	Discuss the potential clinical use of the model and implications for future research.	Discussion para 9
**Other information**				
Supplementary information	21	D;V	Provide information about the availability of supplementary resources, such as study protocol, Web calculator, and data sets.	NA
Funding	22	D;V	Give the source of funding and the role of the funders for the present study.	Funding

*Items relevant only to the development of a prediction model are denoted by D, items relating solely to a validation of a prediction model are denoted by V, and items relating to both are denoted D;V. We recommend using the TRIPOD Checklist in conjunction with the TRIPOD Explanation and Elaboration document.
